# Atrx loss as a promising screening tool for the identification of diffuse midline glioma subtype, H3K27/MAPKinase co-altered

**DOI:** 10.1186/s40478-024-01818-8

**Published:** 2024-06-27

**Authors:** Arnault Tauziède-Espariat, David Castel, Yassine Ajlil, Lucie Auffret, Romain Appay, Cassandra Mariet, Lauren Hasty, Alice Métais, Fabrice Chrétien, Jacques Grill, Pascale Varlet

**Affiliations:** 1 GHU Paris Psychiatrie Neurosciences, Department of Neuropathology, Sainte-Anne Hospital, 1, rue Cabanis, Paris, 75014 France; 2 Paris University France, Paris, 75006 France; 3 U981, Molecular Predictors and New Targets in Oncology, INSERM, Gustave Roussy, Université Paris- Saclay, Villejuif, 94805 France; 4 Univ. Evry, Université Paris-Saclay, Evry, 91000 France; 5 Department of Pathology, APHM La Timone, Marseille, France; 6 Department of Pediatric Oncology, Gustave Roussy, Université Paris-Saclay, Villejuif, 94805 France

Over the last few years, the generalization of stereotactic biopsies for midline tumors and molecular advances has permitted a subdivision of diffuse midline gliomas (DMG), H3K27-altered into five subtypes. These subtypes are based on their molecular characteristics, age and/or location: DMG, H3.3 K27-mutant; DMG, H3.1 or H3.2 K27-mutant; DMG, H3-wildtype, with EZHIP overexpression; DMG, *EGFR*-altered, and very recently DMG, H3K27/MAPK pathway co-altered [1–3]. The last subgroup listed includes DMG with H3K27M mutation (or occasionally an EZHIP overexpression) associated with a MAPK alteration (mainly *FGFR1* or *BRAF* mutations) and is defined by a distinct methylation class [2, 3]. When compared to the classical DMG, H3K27-altered, this group harbors *ATRX* (alpha-thalassemia/mental retardation, X-linked) alterations more frequently than their not-MAPK DMG-H3K27-altered counterparts [2]. Herein, we report on a cohort of DMG H3K27-altered harboring a loss of ATRX expression and describe them in terms of clinical, histopathological, and biological (including DNA-methylation profiling) observations to determine if the loss of ATRX expression may constitute a potential diagnostic tool to facilitate the identification of DMG, H3K27/MAPK co-altered. Cases #2–5, 8, 11, 15 and 16 were previously reported in [2].

From a cohort of 182 pediatric (*n* = 159) and adult (*n* = 23) cases of DMG, H3K27-altered compiled from our center (diagnosed between January 1st 2006 and December 31st 2020), we identified 30 tumors harboring a loss of ATRX expression. Twenty-one cases were found to have sufficient material to perform molecular analyses and were included (located in the thalami *n* = 13, the brainstem *n* = 5, the cerebellum *n* = 2, or the spinal cord *n* = 1). For these cases we performed a comprehensive clinical, histopathological, and molecular evaluation (including digital droplet PCR, RNA-sequencing, next generation sequencing, and FISH analyses), as well as DNA methylation profiling (using the v12.8 of the DKFZ classifier).

**Fig. 1:**
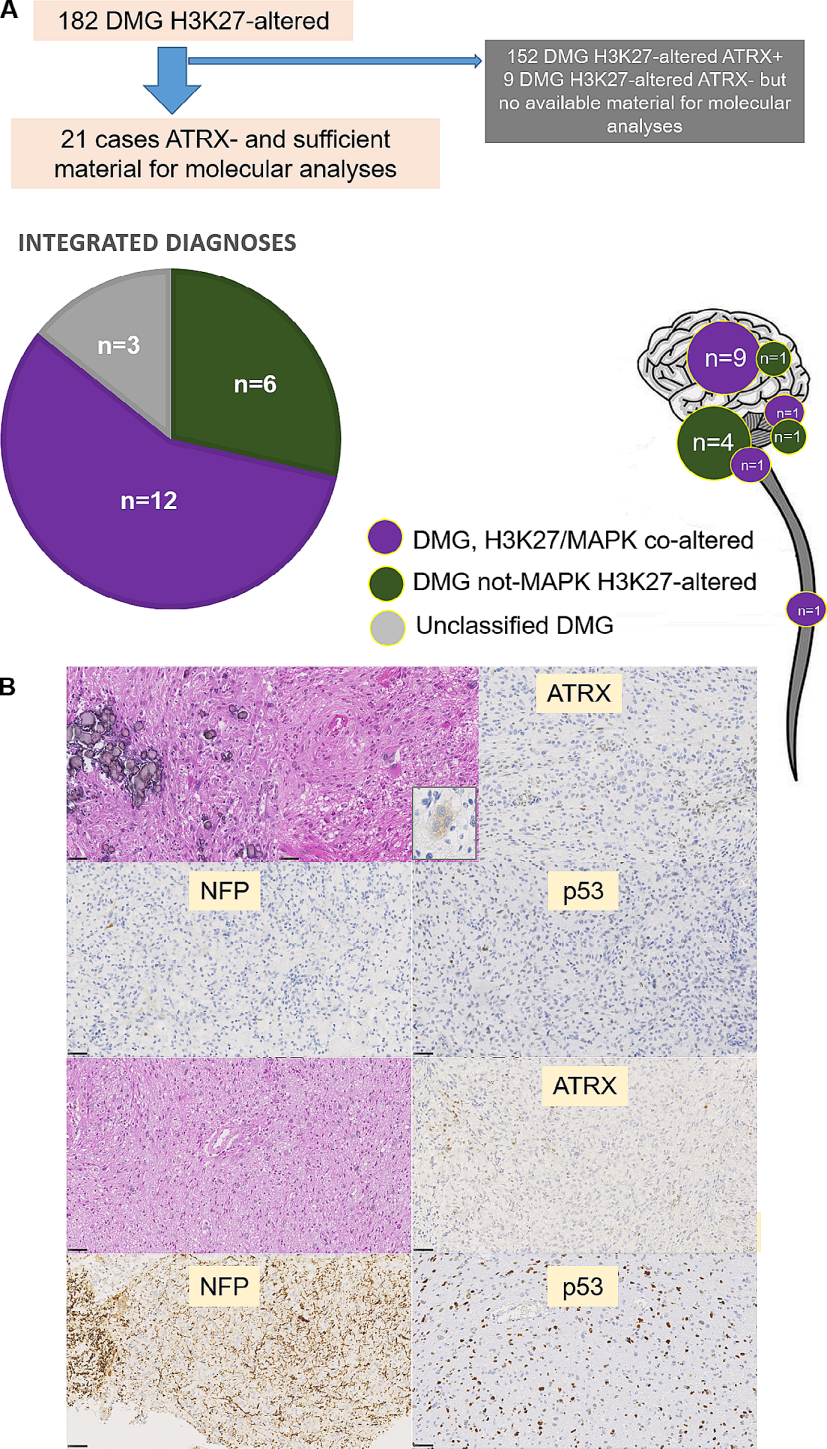
Design of the study and main results. (**A**) Design of the cohort and the distribution of each molecular subgroup of diffuse midline glioma (DMG). (**B**) Main histopathological and immunohistochemical features. DMG, H3K27/MAPK co-altered (two first lines) presented frequently microcalcifications and neuronal features (including binucleated cells immunopositive for synaptophysin, insert). These tumors presented a loss of ATRX expression, a more circumscribed growth pattern using neurofilament protein immunostaining (NFP) and no overexpression of p53. DMG, not MAPK, H3K27-altered showed a diffuse astrocytic proliferation with a loss of ATRX expression. We observed a diffuse pattern and an overexpression of p53. Magnification is x400 for all pictures and black scale bars represent 50 μm

Clinical, histopathological and molecular data from our findings are summarized in supplementary **Tables 1** and illustrated in Fig. 1. The integrative histopathological, genetic and epigenetic analyses, including t-Distributed Stochastic Neighbor Embedding analyses (t-SNE) (Supplementary Fig. 1) segregated the tumors into: twelve DMG, H3K27/MAPK co-altered (57.1%), and six classical DMG, H3K27–altered (with H3K27M mutation) (28.6%). The remaining three DMG, H3K27-altered were unclassified despite all molecular analyses. All DMG, H3K27/MAPK co-altered were located outside the pons and concerned patients aged 12 to 39 years (median: 19). They presented mutations of the *FGFR1* (*n* = 8), *BRAF* (*n* = 1), *PTPN11* (*n* = 1), and *NF1* (*n* = 1) genes. The last case failed to reveal any *FGFR1/BRAF/PTPN11/KRAS* alteration or fusion implicating a MAPK gene, but the *NF1* gene was not explored using molecular analyses. In this group, a *TP53* mutation was observed in only one case. Histopathologically, these tumors frequently presented a circumscribed pattern (10/11 cases with available data), neuronal features (9/11 cases with available data), and microcalcifications (6/12 cases). 75% of patients (9) were dead from their diseases at the end of follow-up (median overall survival of 27 months; ranging from 11 to 86).

Three of the six classical DMG, H3K27-altered were located in the pons and harbored a *TP53* mutation in the five tested cases. All but one case (which was circumscribed), were diffuse, without microcalcifications or neuronal differentiation. All patients having available data were dead from their diseases at the end of follow-up (median overall survival of 13.5 months; ranging from 8 to 26).

The three remaining unclassified cases were monothalamic and presented a MAPK alteration (one *FGFR1* internal tandem duplication, one case with concomitant *FGFR1* and *PTPN11* mutations, and one *BRAF* mutation), but did not cluster within a defined methylation class by t-SNE analysis. They all presented a circumscribed pattern but did not show neuronal features or microcalcifications, and two of the cases harbored a *TP53* mutation. All patients were dead at the end of follow-up (median overall survival of 14 months; ranging from 13 to 17).

DMG, H3K27/MAPK-co-altered have recently been identified as a novel subtype of DMG [2, 3]. As previously described, the results of this series show that these tumors present distinct clinical (affecting mainly adolescents and young adults), radiological (located mainly outside the pons, particularly in the thalami), histopathological (with glioneuronal differentiation, microcalcifications or circumscribed growth), genetic (less frequently harboring a *TP53* mutation) and epigenetic (with a distinct DNA-methylation profile) features [2, 3]. These data may explain why previous reports in the literature have described gangliogliomas [4, 5], diffuse leptomeningeal glioneuronal tumors [6], and more recently, high-grade astrocytomas with piloid features [7], harboring concomitant H3K27M and MAPK alterations. The aforementioned cases may potentially represent different forms of DMG, H3K27/MAPK-co-altered.

While an *ATRX* alteration has been reported in a varying amount of DMGs, it is not yet considered to be a hallmark of DMG, H3K27/MAPK-co-altered (45% of cases) [2], and is rarely present in DMG without MAPK alteration (4 to 13%) [8–12]. Contrary to the previous study, the current work shows that other MAPK alterations than *FGFR1* may be associated with *ATRX* alterations [2]. Alternative lengthening of telomeres (ALT) was shown to be exclusively associated with the H3K27M subgroup in the pons, but rarely due to an *ATRX* alteration [8]. It has recently been suggested that ATRX also play a role in oncogene-induced senescence and may represent another mechanism of impairing oncogene induced senescence in MAPK-altered tumors [13]. Whereas *TP53* mutations are frequently observed in DMG, not-MAPK H3K27-altered, they seem to be very rare in DMG, H3K27/MAPK-co-altered [2]. However, the different subtypes of DMG within the methylation class must be better defined in the future. Indeed, our work shows that three cases presented both H3K27M and MAPK alterations but were not classified by t-SNE analysis (they classed in close vicinity to DMG, H3K27-altered without MAPK alteration).

To summarize, when one considers the prognostic impact for the patients, DMG, H3K27/MAPK-co-altered must be identified by neuropathologists. In this context, a loss of ATRX expression, in conjunction with p53, seems to be good indicator of this novel DMG subtype. This hallmark adds a novel differential diagnostic tool that can be used to distinguish these tumors from other gliomas harboring this loss of expression (astrocytomas, *IDH-*mutant, high-grade astrocytomas with piloid features, diffuse hemispheric gliomas, H3 G34-mutant, and the novel glioneuronal tumor with *ATRX* alteration, kinase fusion and anaplastic features) [1, 7, 14]. These data reinforce the idea that an official diagnosis must integrate all clinical (age of onset), radiological (midline location or hemispheric), histopathological (growth pattern and differentiation), and molecular results.

### Electronic supplementary material

Below is the link to the electronic supplementary material.

Supplementary Material 1

Supplementary Material 2
