# Supplementary figures and images for: Atrx loss as a promising screening tool for the identification of diffuse midline glioma subtype, H3K27/MAPKinase co-altered
Source: Acta Neuropathol Commun. 2024 Jun 27;12:105. doi: 10.1186/s40478-024-01818-8 (PMC11209953; doi:10.1186/s40478-024-01818-8)

tSNE 2

CONTR\_INFLAM

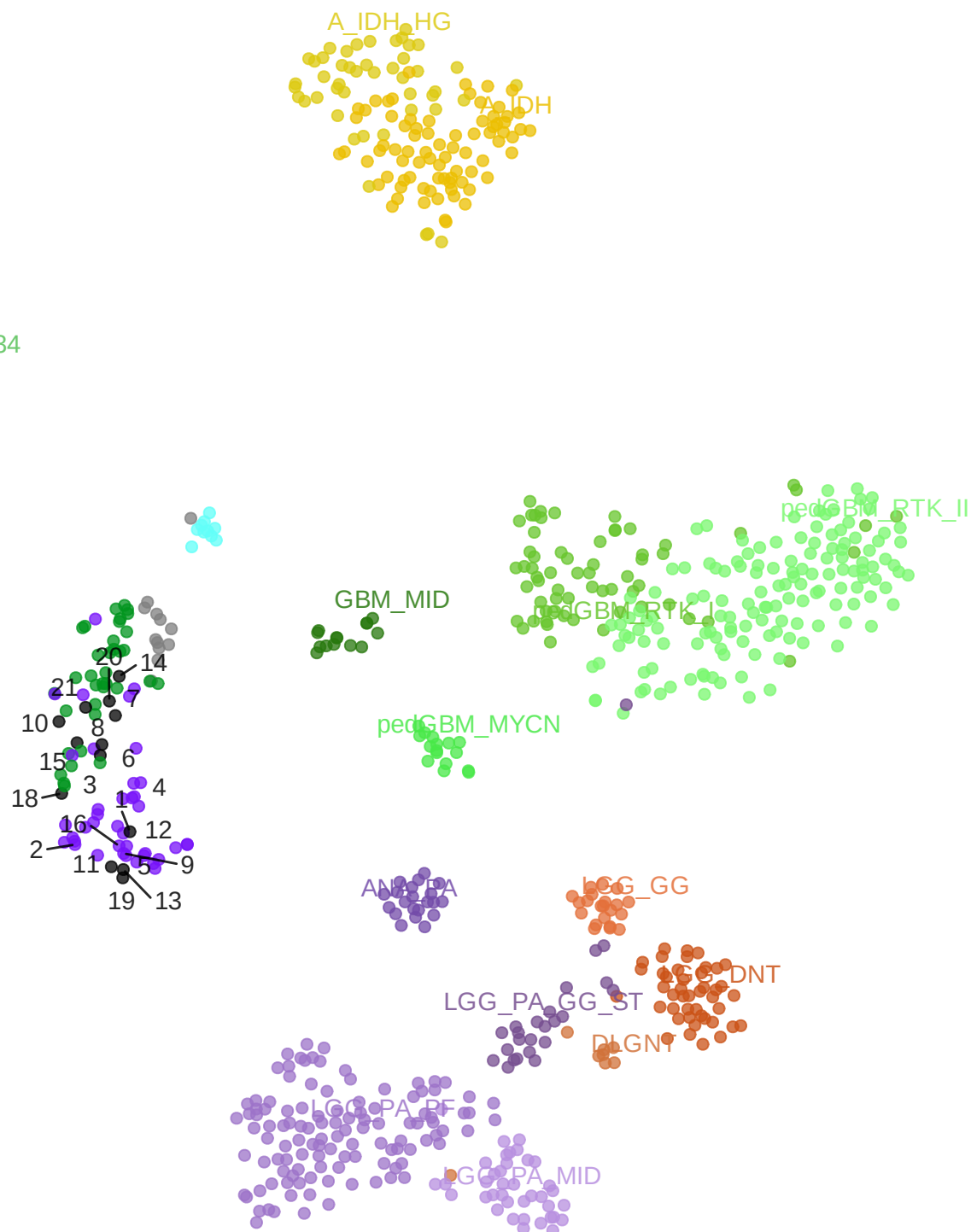

tSNE 1

Classes

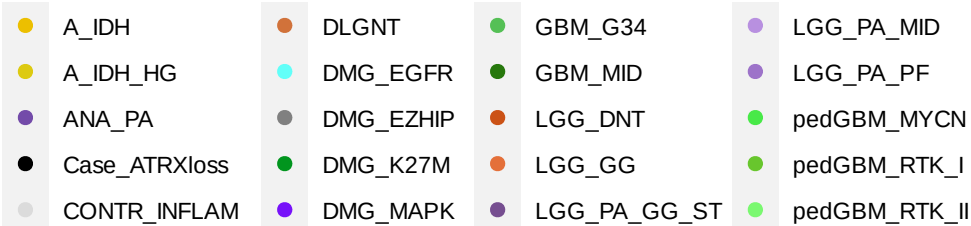

Supplement: Supplementary file 1 — Supplementary Material 1 [file 40478_2024_1818_MOESM1_ESM.pdf]
